# Point-of-care detection and differentiation of anticoagulant therapy - development of thromboelastometry-guided decision-making support algorithms

**DOI:** 10.1186/s12959-021-00313-7

**Published:** 2021-09-07

**Authors:** Simon T. Schäfer, Anne-Christine Otto, Alice-Christin Acevedo, Klaus Görlinger, Steffen Massberg, Tobias Kammerer, Philipp Groene

**Affiliations:** 1 Department of Anaesthesiology, University Hospital Munich, LMU Munich, Munich, Germany; 2 TEM Innovations, Munich, Germany; 3 Department of Internal Medicine I – Cardiology, University Hospital Munich, LMU Munich, Munich, Germany; 4 Department of Anaesthesiology and Intensive Care Medicine, University Hospital of Cologne, Cologne, Germany; 5 Klinikum der Universität München, Ludwig-Maximilians-Universität München, Marchioninistraße 15, 81377 Munich, Germany

**Keywords:** Direct-acting oral anticoagulants, Direct thrombin inhibitors, Direct factor Xa inhibitors, Thromboelastometry

## Abstract

**Background:**

DOAC detection is challenging in emergency situations. Here, we demonstrated recently, that modified thromboelastometric tests can reliably detect and differentiate dabigatran and rivaroxaban. However, whether all DOACs can be detected and differentiated to other coagulopathies is unclear. Therefore, we now tested the hypothesis that a decision tree-based thromboelastometry algorithm enables detection and differentiation of all direct Xa-inhibitors (DXaIs), the direct thrombin inhibitor (DTI) dabigatran, as well as vitamin K antagonists (VKA) and dilutional coagulopathy (DIL) with high accuracy.

**Methods:**

Following ethics committee approval (No 17–525-4), and registration by the German clinical trials database we conducted a prospective observational trial including 50 anticoagulated patients (*n* = 10 of either DOAC/VKA) and 20 healthy volunteers. Blood was drawn independent of last intake of coagulation inhibitor. Healthy volunteers served as controls and their blood was diluted to simulate a 50% dilution in vitro. Standard (extrinsic coagulation assay, fibrinogen assay, etc.) and modified thromboelastometric tests (ecarin assay and extrinsic coagulation assay with low tissue factor) were performed. Statistical analyzes included a decision tree analyzes, with depiction of accuracy, sensitivity and specificity, as well as receiver-operating-characteristics (ROC) curve analysis including optimal cut-off values (Youden-Index).

**Results:**

First, standard thromboelastometric tests allow a good differentiation between DOACs and VKA, DIL and controls, however they fail to differentiate DXaIs, DTIs and VKAs reliably resulting in an overall accuracy of 78%. Second, adding modified thromboelastometric tests, 9/10 DTI and 28/30 DXaI patients were detected, resulting in an overall accuracy of 94%. Complex decision trees even increased overall accuracy to 98%. ROC curve analyses confirm the decision-tree-based results showing high sensitivity and specificity for detection and differentiation of DTI, DXaIs, VKA, DIL, and controls.

**Conclusions:**

Decision tree-based machine-learning algorithms using standard and modified thromboelastometric tests allow reliable detection of DTI and DXaIs, and differentiation to VKA, DIL and controls.

**Trial registration:**

Clinical trial number: German clinical trials database ID: DRKS00015704.

**Supplementary Information:**

The online version contains supplementary material available at 10.1186/s12959-021-00313-7.

## Background

Direct oral anticoagulants (DOACs) are common drugs for prevention and therapy of thromboembolic events. Vitamin K antagonists (VKA) were considered the gold standard for oral anticoagulation until DOACs came on the market in 2008. Actually, DOACs are of predominantly used since several studies have shown a better risk-benefit profile for DOACs compared to VKA [1–4]. Furthermore, in contrast to VKAs, which require routine INR testing, routine drug monitoring for DOACs is not required since pharmacokinetics are predictable [5]. However, emergency situations, including stroke or acute bleeding situations, require exact and fast tests to detect whether a patient has relevant DOAC plasma concentrations, especially if the patient is unconscious and drug history is not available [6–9].

Furthermore, it is important to differentiate the two classes of DOACs (direct factor Xa inhibitors (DXaIs) and direct thrombin inhibitors (DTIs)), as well as DOACs from VKAs and dilutional coagulopathy (DIL). This is essential to timely initiate the adequate hemostatic therapy in cases of bleeding or emergency interventions [5, 10, 11].

Thus, thromboelastometric tests, performed at the point-of-care, could be a helpful approach for the detection of DOACs in the emergency room [12–16]. In this regard, standard thromboelastometric tests are poor in detection of DXaIs at low concentrations or even differentiation between DXAIs on the one hand, and DTI and VKAs on the other hand [17]. To improve DOAC-detection we have recently shown that a set of modified thromboelastometric assays can differentiate rivaroxaban and dabigatran [18, 19]. However, it is unclear whether a set of standard and modified thromboelastometric tests allows detection of the further available DXaIs (apixaban, edoxaban) and differentiation to VKAs or DIL, respectively.

Therefore, we tested the hypotheses, that a set of standard and modified thromboelastometric tests allows detection and differentiation of DXaIs (rivaroxaban, apixaban, and edoxaban), DTI (dabigatran), VKAs (phenprocoumon) and DIL using a decision tree-based algorithm.

## Methods

The study was approved by the Ludwig-Maximilian-University’s ethics committee (No 17–525-4), registered by the German clinical trials database (ID: DRKS00015704, date: 10/05/2018) and performed in accordance with the Declaration of Helsinki. Written informed consent was obtained from patients and healthy volunteers prior to study inclusion.

### In-vivo prospective observational trial

50 patients with constant intake (> 7 days) of DOACs (dabigatran, rivaroxaban, apixaban, edoxaban) or vitamin K antagonists (phenprocoumon) were included in this prospective observational trial (*n* = 10 per substance). Additionally, 20 healthy volunteers without intake of any anticoagulant were included as control group.

Blood was taken once, independent of last intake of medication. This approach was chosen to simulate the clinical situation of emergency patients for whom the time point of last medication intake varies. Exclusion criteria were age under 18, intake of two or more anticoagulants (e.g. dual antiplatelet therapy and DOAC), other known coagulation disorders (e.g. von Willebrand’s disease) or myelodysplastic syndrome as well as patient’s denial. Platelet inhibitors as acetylsalicylic acid or clopidogrel were allowed. Every blood sample was immediately processed and all thromboelastometric and standard laboratory tests were performed within two hours.

#### In-vitro dilutional coagulopathy

We simulated dilutional coagulopathy using blood samples of healthy volunteers. First, citrated blood was diluted by 50% with citrated saline 0.9% (dilution 1).

Second, to simulate a more clinically relevant situation we added washed packed red blood cells to 50% diluted blood (dilution 2): In detail, we centrifuged (2000 g; 10 min) 5 ml citrated blood to obtain packed red blood cells. After centrifugation 2 ml of the packed red blood cells were diluted with 2 ml citrated saline 0.9% and centrifuged again to clean it from plasma. Then 1 ml of the washed packed red blood cells was mixed with 4 ml of the 50% dilution (2 ml citrated blood and 2 ml citrated saline 0.9%; dilution 2), resulting in a median hemoglobin concentration of 9.9 g dl-1 (interquartile range (IQR), 9.1/10.3) (supplemental Table 1).

#### Thromboelastometry

Standard thromboelastometric tests (NATEM, EXTEM, FIBTEM, INTEM, HEPTEM) were performed for each sample using ROTEM delta analyzers (TEM Innovations GmbH, Munich, Germany) in accordance to manufacturer’s protocol [20]. NATEM represents spontaneous blood coagulation without coagulation activators after the blood sample is recalcificated. In EXTEM and FIBTEM coagulation is initiated by tissue factor (representing the extrinsic pathway of coagulation by which the name comes about) after the sample is recalcificated. FIBTEM additionally includes cytochalasin D which blocks platelet contribution to clot firmness. Furthermore, in EXTEM and FIBTEM heparin is blocked by Polybrene. INTEM and HEPTEM represent the intrinsic pathway of coagulation by which the name comes about for INTEM, and coagulation is initiated using elagic acid. INTEM is Heparin sensitive, whereas HEPTEM includes heparinase to block heparin. In addition to these standard tests, we performed modified thromboelastometric tests (TFTEM and ECATEM) as shown previously [21]. TFTEM contains 90% less tissue factor compared to EXTEM, and thus is more sensitive to changes in thrombin generation, e.g. due to the effects of DTIs, DXaIs, vitamin K antagonists and even hemophilia due to lower coagulation triggering [12, 22–24].

ECATEM uses the snake venom ecarin. Ecarin directly converts prothrombin to meizothrombin which converts fibrinogen to fibrin. Meizothrombin has a lower activity compared to thrombin and is inhibited by direct thrombin inhibitors such as hirudin, argatroban, bivalirudin and dabigatran but not by heparin [12, 25–27]. ECATEM is insensitive to any changes in the activity of factor V, VII, VIII, IX, X, XI, or XII, and thus might be unaltered with DXaIs but sensitive to DTIs [28, 29].

Standard parameters provided by the system are clotting time (CT; time from initiation of the clotting process to a 2-mm clot amplitude), clot formation time (CFT; CT until a clot amplitude of 20 mm is reached), A5 (clot amplitude 5 min after CT), A10 (clot amplitude 10 min after CT) or maximum clot firmness (MCF; the maximum amplitude of the clot). Additionally, parameters demonstrating clot lysis as maximum lysis or clot lysis index are provided. Clotting time (CT) is affected by each anticoagulant by blocking the appropriate synthesis of several (phenprocoumon) or inhibition of specific coagulation factors (DOACs) as well as by dilution. This results in a prolonged clotting time. A5 and A10 are time dependent measures of clot firmness. Dependent on the extent to which the individual coagulation factors are influenced, the development of clot firmness can be decreased, particularly if fibrinogen is decreased in dilutional coagulopathy.

#### Standard laboratory tests

Standard laboratory tests were measured by the Ludwig-Maximilians-University institute for laboratory medicine, according to institutional rules and regulations.

Standard coagulation variables including international normalized ratio (INR) (Thromborel S, Siemens Healthcare GmbH, Erlangen, Germany), thrombin time (TT) (Berichrom Thrombinreagenz, Siemens Healthcare GmbH, Erlangen, Germany), activated partial thromboplastin time (aPTT) (Actin FSL, Siemens Healthcare GmbH, Erlangen, Germany) and blood count were performed. Substance specific and calibrated anti-Xa/anti-IIa tests were performed using Hemoclot Thrombin inhibitors test (Hyphen Biomed, Neuville-sur-Oise, France) and Coamatic Heparin test (Haemochrom Diagnostica GmbH, Essen, Germany).

#### Statistical analysis

Statistical analysis was performed using SPSS, Version 25 (IBM, Armonk, USA) and Graph Pad Prism 8 (GraphPad Software Inc., La Jolla, USA). To focus on clinically relevant situations, we grouped particular substances for the analysis based on the mode of action of the drug. Thus, we summarized rivaroxaban, apixaban and edoxaban as DXaIs and the two in-vitro dilutions as dilutional coagulopathy (DIL).

To mount a decision tree for the differentiation of coagulopathies we used SPSS Decision Tree, Version 25 (IBM, Armonk, USA). For analysis, we entered all thromboelastometric variables as well as estimated variables consisting of the single thromboelastometric variables and performed the “classification and regression” (CART) mode of SPSS decision tree (twoing, minimal improvement 0.05, prune = 1) [30]. This method analysis the optimal discriminants for a model with the maximum accuracy for the prediction of a categorical dependent value.

Additionally, we performed receiver operating characteristic (ROC) curve analyzes with Youden-index to support and verify the established decision tree.

The novel methods and systems described in this publication are covered in a pending U.S. patent application.

## Results

We enclosed 50 anticoagulated patients and 20 healthy volunteers to this prospective observational trial. Detailed patients’ and volunteers’ characteristics are displayed in supplemental Table 1. Results of all thromboelastometric (CT NATEM, CT EXTEM, CT INTEM, CT FIBTEM, CT TFTEM, CT ECATEM, CT HEPTEM, A5 NATEM, A5 EXTEM, A5 FIBTEM, A5 INTEM, A5 HEPTEM, A5 TFTEM, A5 ECATEM) and standard laboratory tests (INR, aPTT, TT, DOAC plasma concentration measured by anti-Xa and anti-IIa activity) stratified for the specific anticoagulant, are displayed in supplemental Table 2.

**Fig. 1:**
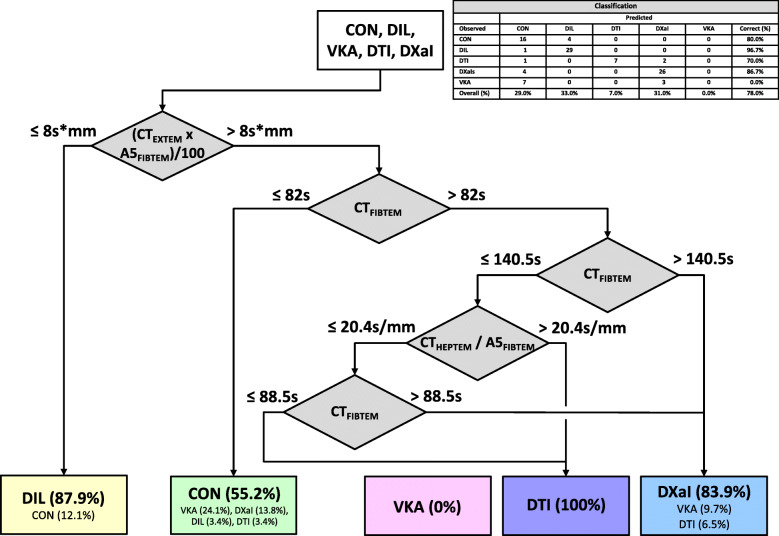
Decision Tree 1 (DT1) includes standard thromboelastometric tests (EXTEM, FIBTEM, HEPTEM, INTEM). Overall classification accuracy is 78%. Percentage in boxes display the portion of included samples at this stage. CON: control; DIL: dilutional coagulopathy; VKA: vitamin K antagonist (phenprocoumon); DTI: direct thrombin inhibitor (dabigatran); DXaI: direct factor Xa inhibitors (apixaban, rivaroxaban and edoxaban)

In a first step we established a decision tree (DT1; Fig. 1) based on standard thromboelastometric tests according to the “complete + hep” cartridge of the ROTEM *sigma* including EXTEM, FIBTEM, INTEM and HEPTEM to differentiate the different anticoagulants, controls and dilution samples. The final version only uses three tests (EXTEM, FIBTEM, HEPTEM) and estimated variables (indices) consisting of different single variables like the product of CT EXTEM and A5 FIBTEM. DT1 classifies 78% of the samples correctly. In detail, 80% of the controls and 96.7% of the samples with dilutional coagulopathy are classified correctly. In contrast anticoagulant differentiation is more difficult using only standard thromboelastometric tests. Only 70% of the DTI samples, 86.7% of the DXaI samples and none of the VKA samples were assigned correctly (Fig. 1).

**Fig. 2:**
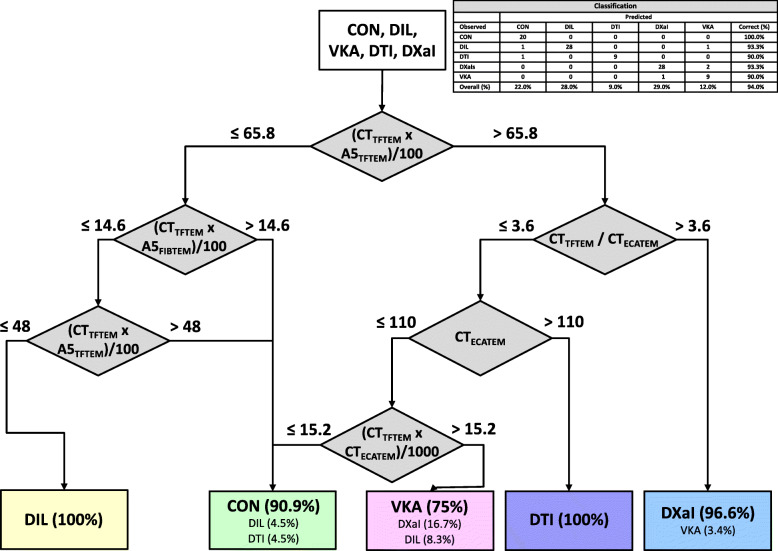
Decision Tree 2 (DT2) includes standard and new thromboelastometric tests (FIBTEM, TFTEM, ECATEM). Overall classification accuracy is 94%. Percentage in boxes display the portion of included samples at this stage. CON: control; DIL: dilutional coagulopathy; VKA: vitamin K antagonist (phenprocoumon); DTI: direct thrombin inhibitor (dabigatran); DXaI: direct factor Xa inhibitors (apixaban, rivaroxaban and edoxaban)

In a second step we established a decision tree (DT2; Fig. 2) including the same standard thromboelastometric tests and variables but included the new evaluated ECATEM and TFTEM test which are more specific for direct thrombin inhibitors (ECATEM) and anticoagulants in general (TFTEM). Furthermore, we limited the maximum tree depth. We calculated two version of the decision tree in a first round. One used EXTEM, FIBTEM, TFTEM and ECATEM and the second run was done with HEPTEM, FIBTEM, TFTEM and ECATEM according to the situation that a ROTEM analyzer only offers four channels and it would be unrealistic for clinical use to include more than four tests. Both versions offered an overall accuracy of 94% and no differences in the detection of the single substances. In detail, DT2 as well only uses three tests (FIBTEM, TFTEM, ECATEM) and some estimated variables (indices) in its final version. In a first step it differentiates all samples with oral anticoagulants from almost all controls and dilutional coagulopathy by the product of CT TFTEM and A5 TFTEM. The oral anticoagulants are differentiated using variables of TFTEM and ECATEM (Ratio CT TFTEM/CT ECATEM, CT ECATEM, and product of CT TFTEM and CT ECATEM) in the following steps (Fig. 2). Summarized 90% of the samples with DTI and VKA are classified correctly and 93.3% of the DXaI samples. On the other hand, controls and dilutional coagulopathy are differentiated using variables of TFTEM and FIBTEM (product of CT TFTEM and A5 FIBTEM and product of CT TFTEM and A5 TFTEM). This leads to an overall correctness of 100% in detection of control samples and 93.3% in detection of dilutional coagulopathy (Fig. 2).

**Fig. 3:**
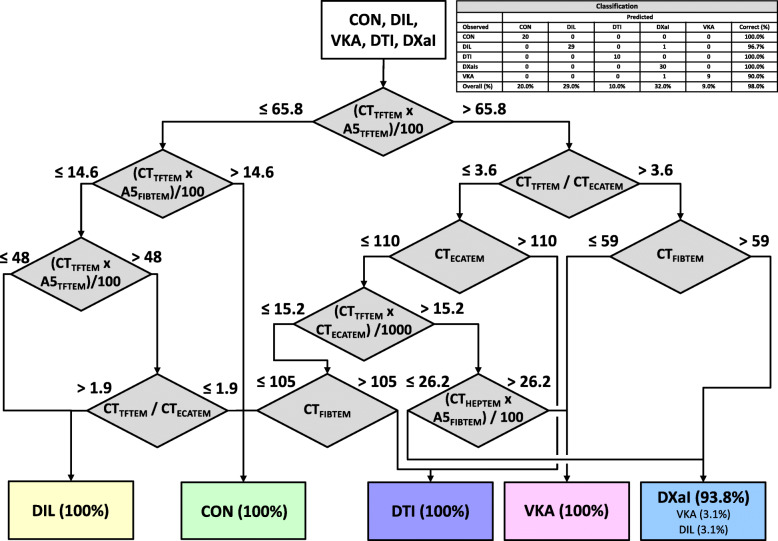
Decision Tree 3 (DT3) includes standard and new thromboelastometric tests (FIBTEM, HEPTEM, TFTEM, ECATEM). Overall classification accuracy is 98%. Percentage in boxes display the portion of included samples at this stage. CON: control; DIL: dilutional coagulopathy; VKA: vitamin K antagonist (phenprocoumon); DTI: direct thrombin inhibitor (dabigatran); DXaI: direct factor Xa inhibitors (apixaban, rivaroxaban and edoxaban)

In a third step we used all the tests (variables) of DT2 but did not limit the maximum tree depth which resulted in a more complex and more branched decision tree. According to step 2 we calculated two versions in a first round. Overall accuracy was 98% for both versions but including HEPTEM instead of EXTEM lead to a more precisely discrimination of oral anticoagulants. In this version no oral anticoagulant was misleadingly classified as control or dilutional coagulopathy. Therefore, we used the four tests HEPTEM, FIBTEM, TFTEM and ECATEM to establish the final versions of our DTs (Fig. 3). In detail, DT3 uses four tests (FIBTEM, HEPTEM, TFTEM, ECATEM) and some estimated variables (indices). DT2 and DT3 are comparable in the first branches. DT3 as well separates oral anticoagulants from controls and dilutional coagulopathy in a first step (product of CT TFTEM and A5 TFTEM) and differentiates the anticoagulants then using variables of TFTEM and ECATEM. The difference is that DT3 then uses FIBTEM and estimated variables including CT HEPTEM and A5 FIBTEM to reach more accuracy in the detection of the DTI (100%; Fig. 2[KG3]). Controls and dilutional coagulopathy are detected more precisely by including the ratio between CT TFTEM and CT ECATEM (controls: 100% and DIL: 96.7%).

To support the decisions made by the program to establish the different versions of the decision trees we additionally calculated ROC curve analyzes. For every node which is made in one of the DTs we calculated the specific ROC analysis; e.g. the differentiation between oral anticoagulants and controls plus dilutional coagulopathy which is the first node of DT2 and DT3. This ROC curve analysis shows an area under the curve (AUC) of 0.991 (SE 0.006) with a sensitivity of 100% and a specificity of 92%. Another key node is the differentiation between DXaIs on the one hand, and DTI plus VKA on the other hand, which is made by the ratio between CT TFTEM and CT ECATEM. ROC curve analysis here shows an AUC of 0.970 (SE 0.021) with a sensitivity of 93% and a specificity of 90%. For the differentiation between controls and dilutional coagulopathy the product of CT TFTEM and A5 FIBTEM is essential. Here ROC curve analysis shows an AUC of 0.920 (SE 0.0519) with a sensitivity of 90% and a specificity of 93%. All other ROC curve analyzes are displayed in Table 1.

**Table 1:** ROC Curve Analyzes

Discrimination	Variable	AUC (SE)	***P***-value	Optimum cutoff	Sensitivity	Specificity
**CON + DIL vs. OAC**	CT_EXTEM_	0.915 (0.027)	< 0.001	91.5	0.74	0.96
	CT_FIBTEM_	0.858 (0.040)	< 0.001	75.5	0.82	0.84
	**CT**_**TFTEM**_	**0.991 (0.006)**	**< 0.001**	**194.5**	**0.94**	**0.98**
	A5_FIBTEM_	0.814 (0.042)	< 0.001	8.5	0.98	0.54
	A5_TFTEM_	0.714 (0.051)	< 0.001	36.5	0.88	0.48
	**(CT**_**EXTEM**_**x A5**_**FIBTEM**_**) / 100**	**0.952 (0.018)**	**< 0.001**	**9.3**	**0.96**	**0.80**
	**(CT**_**TFTEM**_**x A5**_**FIBTEM**_**) / 100**	**0.972 (0.013)**	**< 0.001**	**20.9**	**0.96**	**0.86**
	**(CT**_**TFTEM**_**x A5**_**TFTEM**_**) / 100**	**0.991 (0.006)**	**< 0.001**	**65.8**	**1.00**	**0.92**
	**(CT**_**TFTEM**_**x CT**_**HEPTEM**_**) / 1000**	**0.984 (0.009)**	**< 0.001**	**36.5**	**0.94**	**0.94**
**CON vs. DIL**	CT_EXTEM_	0.380 (0.079)	0.154	58.5	0.90	0.20
	CT_FIBTEM_	0.482 (0.084)	0.828	63.0	0.75	0.37
	**A5**_**EXTEM**_	**0.934 (0.350)**	**< 0.001**	**38.5**	**0.90**	**0.90**
	**A5**_**FIBTEM**_	**0.919 (0.050)**	**< 0.001**	**11.5**	**0.85**	**0.90**
	**(CT**_**TFTEM**_**x A5**_**FIBTEM**_**) / 100**	**0.920 (0.051)**	**< 0.001**	**13.4**	**0.90**	**0.93**
	(CT_TFTEM_ x A5_TFTEM_) / 100	0.908 (0.056)	< 0.001	47.3	0.90	0.93
**DXaIs vs. DTI + VKA**	***CT***_***EXTEM***_	0.782 (0.067)	0.001	129.0	0.70	0.85
	***CT***_***FIBTEM***_	0.827 (0.061)	< 0.001	140.5	0.70	0.95
	***CT***_***HEPTEM***_	0.462 (0.880)	0.649	167.5	1.00	0.10
	***CT***_***TFTEM***_	**0.950 (0.033)**	**< 0.001**	**355.5**	**0.90**	**0.95**
	***CT***_***ECATEM***_	0.288 (0.083)	0.012	49.0	1.00	0.05
	***CT-ratio***_***TFTEM/ECATEM***_	**0.970 (0.021)**	**< 0.001**	**3.56**	**0.93**	**0.90**
	***CT***_***HEPTEM***_***/A5***_***FIBTEM***_	0.450 (0.850)	0.552	15.8	0.50	0.60
	***(CT***_***TFTEM***_***x CT***_***ECATEM***_***) / 1000***	0.717 (0.079)	0.010	24.3	0.93	0.50
	(CT_HEPTEM_ x A5_FIBTEM_) / 100	0.512 (0.083)	0.890	54.6	0.40	0.85
**DTI vs. DXaIs + VKA**	***CT***_***EXTEM***_	0.409 (0.086)	0.376	86.0	0.90	0.25
	***CT***_***FIBTEM***_	0.388 (0.083)	0.275	82.0	0.90	0.28
	***CT***_***HEPTEM***_	0.746 (0.093)	0.017	263.5	0.70	0.45
	***CT***_***TFTEM***_	0.124 (0.051)	< 0.001	149.0	1.00	0.00
	***CT***_***ECATEM***_	**0.900 (0.095)**	**< 0.001**	**111.5**	**0.90**	**1.00**
	***CT-ratio***_***TFTEM/ECATEM***_	0.025 (0.026)	< 0.001	9.9	0.00	1.00
	***CT***_***HEPTEM***_***/A5***_***FIBTEM***_	0.740 (0.078)	0.020	12.7	1.00	0.48
	***(CT***_***TFTEM***_***x CT***_***ECATEM***_***) /1000***	0.552 (0.109)	0.611	33.3	0.70	0.53
	(CT_HEPTEM_ x A5_FIBTEM_) / 100	0.565 (0.095)	0528	33.8	0.80	0.43
VKA vs. DXaIs+DTI	***CT***_***EXTEM***_	0.169 (0.056)	0.001	67.0	1.00	0.05
	***CT***_***FIBTEM***_	0.123 (0.048)	< 0.001	56.0	1.00	0.00
	***CT***_***HEPTEM***_	0.311 (0.095)	0.067	438.5	0.10	0.98
	***CT***_***TFTEM***_	0.201 (0.060)	0.004	190.0	1.00	0.08
	***CT***_***ECATEM***_	0.419 (0.089)	0.431	67.5	1.00	0.08
	***CT-ratio***_***TFTEM/ECATEM***_	0.270 (0.069)	0.026	1.76	1.00	0.23
	***CT***_***HEPTEM***_***/A5***_***FIBTEM***_	0.335 (0.980)	0.109	38.0	0.10	1.00
	***(CT***_***TFTEM***_***x CT***_***ECATEM***_***) /1000***	0.123 (0.050)	< 0.001	15.5	1.00	0.05
	(CT_HEPTEM_ x A5_FIBTEM_) / 100	0.418 (0.083)	0.423	24.3	1.00	0.15
**DTI vs. VKA**	CT_HEPTEM_	0.810 (0.107)	0.190	216.5	0.90	0.70
	**CT**_**ECATEM**_	**0.900 (0.095)**	**0.002**	**110.0**	**0.90**	**1.00**
	**(CT**_**ECATEM**_**x CT**_**HEPTEM**_**) / 1000**	**0.910 (0.064)**	**0.002**	**27.7**	**0.80**	**0.90**
**DTI vs. DXaIs**	**CT**_**ECATEM**_	**0.900 (0.095)**	**< 0.001**	**111.5**	**0.90**	**1.00**
	**CT-ratio**_**TFTEM/ECATEM**_	**0.993 (0.009)**	**< 0.001**	**3.56**	**0.93**	**1.00**
	(CT_ECATEM_ x CT_HEPTEM_) / 1000	0.880 (0.073)	< 0.001	37.9	0.70	1.00
**DXaIs vs. VKA**	CT_EXTEM_	0.845 (0.061)	0.001	125.5	0.73	1.00
	CT_FIBTEM_	0.897 (0.050)	< 0.001	106.5	0.83	1.00
	CT_HEPTEM_	0.648 (0.102)	0.165	222.5	0.60	0.80
	**CT**_**TFTEM**_	**0.948 (0.036)**	**< 0.001**	**355.5**	**0.90**	**1.00**
	**CT-ratio**_**TFTEM/ECATEM**_	**0.947 (0.037)**	**< 0.001**	**3.60**	**0.93**	**0.90**
	(CT_TFTEM_ x CT_HEPTEM_) / 1000	0.910 (0.047)	< 0.001	67.6	0.90	0.80

ROC, receiver operating characteristics; AUC, area under the curve; SE, standard error; CT, coagulation time; CON, controls; DIL, dilutional coagulopathy; OAC, oral anticoagulants; DXaI, direct factor Xa inhibitors (apixaban, edoxaban, rivaroxaban); DTI, direct thrombin inhibitor (dabigatran); VKA, vitamin K-antagonist (phenprocoumon)

A graphical overview on the different variables used can be seen in supplemental Fig. 1 which displays the distribution of all samples in CT FIBTEM, CT TFTEM, CT ECATEM, A5 TFTEM, ratio between CT TFTEM and CT ECATEM and A5 FIBTEM.

## Discussion

In this prospective observational trial, we show that a set of standard and modified thromboelastometric tests detect and differentiate DXaIs (rivaroxaban, apixaban, and edoxaban), DTI (dabigatran), VKAs (phenprocoumon) and DIL using a decision tree-based algorithm. We show that the accuracy of detection and differentiation of oral anticoagulants improve from 78% (DT1) using standard thromboelastometric tests alone to 94% (DT2) by the additional use of two new modified thromboelastometric tests (TFTEM and ECATEM) from 78% (DT1) to 94% (DT2). Furthermore, accuracy could be improved from 94% (DT2) to 98% (DT3) by a more complex decision-tree algorithm with unlimited maximum tree depth (here maximum five nodes).

This new, bedside available, thromboelastometric approach can help the clinician to make rapid, accurate and specific treatment decisions in case of acute bleeding or stroke within a few minutes.

Recent research focuses on rapid detection of oral anticoagulants since more and more patients are on these drugs due to a growing older population. Up to date, detection and differentiation of oral anticoagulants, especially DOACs is challenging. Most standard laboratory tests are either unspecific or too sensitive for DOAC-detection, like TT for DTI. Solely, ecarin time allows specific detection and estimation of the DTI dabigatran. Additionally, substance-specific, calibrated tests are able to quantify DOAC plasma concentrations. As these tests are calibrated for a specific drug, they can only be used if the drug taken is known. This limits the use of those tests in several emergency situations due to lacking information about drug history.

Due to the long turnaround time of calibrated DOAC tests, they are not suitable for emergency situations such as intracranial hemorrhage, stroke and severe bleeding in trauma or traumatic brain injury [12, 31–33]. Furthermore, the tests are not available at all hospitals 24/7. Unfortunately, measurement of anti-Xa activity showed heterogeneous results and especially detection of DOAC plasma concentrations < 50 ng ml-1 and > 300 ng ml-1 is difficult [34–36]. Some of these assays showed falsely high plasma concentrations even in samples of controls without DOAC intake.

Recently, determination of DOAC urine concentration became available [37]. Even with this test, providing results within minutes, its usefulness is limited in renal insufficiency as well as in anuria in severe shock and does not allow for differentiation from VKA or DIL. INR is considered as the gold standard for VKA monitoring, and routine checks of coagulation status are done by this test. Nevertheless, turnaround time is longer than thromboelastometry and INR on its own is not able to differentiate VKA effects from other anticoagulants due to the fact that DOACs increase INR, too [12, 38]. This applies to the other standard laboratory tests such as partial thromboplastin time as well [12, 38].

Therefore, thromboelastometric tools came into the focus for detection and differentiation of DOACs, as they are available at the point-of-care and turnaround time is around 10–15 min [39, 40]. Initially, standard thromboelastometric assays like EXTEM or INTEM were used [14–16]. However, Seyve et al. demonstrated that these tests are not specific and not sensitive enough for apixaban detection, as CT INTEM values for apixaban stayed within the normal range even with supratherapeutic plasma concentrations [17]. Thus, standard thromboelastometric tests are of limited value for DXaI detection.

Using modified assays with lower amounts of tissue factor and ecarin-based assays led to more sensitive detection compared to standard tests [24, 27, 41]. Further improvements and combination of these tests allow differentiation of rivaroxaban and dabigatran as Vedovati et al. and we have demonstrated recently [18, 19]. Two recently published studies with the TEG 6 s system successfully evaluated in-vivo samples to detect patients on DOAC treatment and to differentiate controls, patients on DXaIs and patients on dabigatran [42, 43]. Unfortunately, none of the prior studies evaluated whether a differentiation between healthy controls, DXaIs, DTIs, VKA or DIL is possible, and which now has been demonstrated by this study. This aspect is of particular interest and clinical relevance since each anticoagulant requires specific therapeutic interventions. The now available, specific, but very expensive DOAC antidotes also need evidence-based and rational prescription in emergency situations. Especially, the use of the new antagonist andexanet alfa requires knowledge about last intake and dosage of FXa-inhibitors due to two different dosing regimens dependent on this information [10]. In this regard a decision tree-based algorithm using thromboelastometry can provide essential information for emergency treatment within minutes.

Further studies with bigger patient populations have to validate the cut-off values to detect the specific drugs and the correlation between coagulation time prolongation and DOAC plasma concentration on the one hand and bleeding on the other hand.

We present different versions of decision trees in this manuscript. In a first step we decided to evaluate standard thromboelastometric tests only to see if the addition of modified tests significantly improve the detection of oral anticoagulants and their differentiation. We clearly see that the modified tests improve accuracy of detection and differentiation. One difficulty of this approach was to decide which of the standard tests can be used in addition to the modified tests, especially considering that ROTEM only provides four channels for testing. We concentrated on the standard tests used in the cartridges complete + hep of the fully automated ROTEM sigma which include EXTEM, FIBTEM, INTEM, and HEPTEM. Based on its worldwide accepted usefulness in detecting hypofibrinogenemia and to predict bleeding and transfusion, we decided to include FIBTEM for detection of dilutional coagulopathy and differentiation from healthy controls and patients treated with oral anticoagulants [44–46]. The diagnostic performance of CT FIBTEM was at least as high as CT EXTEM for the detection of oral anticoagulants in our ROC curve analyses (Table 1) which is in-line with the data published by other authors [16]. Furthermore, FIBTEM provides reliable clot firmness results even at direct thrombin inhibitor concentrations which significantly impacts plasma fibrinogen determinations with the Clauss method [47]. Therefore, FIBTEM is a key assay in patients suspected to be treated with DTIs. Considering these aspects, we had to choose between EXTEM, INTEM and HEPTEM. EXTEM provides almost the same CT-results as FIBTEM but includes the impact of platelets to the clot. In contrast to INTEM, HEPTEM provides the advantage of eliminating any potential heparin effects and therefore any interference between unfractionated or low molecular weight heparin with DOACs. EXTEM and FIBTEM tests include polybrene for heparin neutralization to avoid an interference with heparin in these assays, too. Accordingly, we chose HEPTEM over INTEM and then created different versions of the decision tree either using HEPTEM or EXTEM. Overall detection rate was the same but using HEPTEM improved differentiation of vitamin K-antagonists versus DXaIs. For daily practice and emergency treatment, it is crucial to detect anticoagulants correctly and using HEPTEM improved this aspect compared to the versions with EXTEM.

Another aspect we had to consider was the complexity of the algorithm. Therefore, we limited the depth of the tree in version 2 (Fig. 2). This version can be used without computer support. Nevertheless, we tried to find an algorithm not limited by complexity but providing the highest accuracy possible based on the selected standard and modified thromboelastometric tests as shown in DT3 (Fig. 3). The use of this decision tree presupposes a software support which can be integrated in the ROTEM device or a corresponding middleware after validation in further studies.

Our study has several limitations. First, dilutional coagulopathy was simulated in-vitro, and two standardized conditions were used. In contrast, trauma-induced coagulopathy is much more complex, highly variable and influenced besides dilution by additional factors such as endothelial integrity, glycocalyx shedding, endogenous heparinoids, temperature acidosis and platelet dysfunction [48]. This was not analyzed in our study. However, the in-vitro dilutional coagulopathy models were chosen as we could standardize the DIL group using this approach in this algorithm development study. In this first approach to establish an algorithm we wanted to find out, whether our algorithm can discriminate patients on different oral anticoagulants from controls as well as dilutional coagulopathy since this is important in the clinical setting of patients admitted to the emergency room, e.g., after trauma. As the next step, we will ascertain the algorithm in a validation study recruiting patients admitted to the emergency room after trauma or other clinical settings associated with bleeding.

Second, since we analyzed samples independently of the last intake the plasma concentrations may not have covered the entire range possible. However, the included samples show a widespread distribution of plasma concentrations. Anyway, sample size was small and especially more samples with low plasma concentrations have to be evaluated to validate the algorithm and its sensitivity and specificity. Further studies evaluating more samples according to the Clinical and Laboratory Standards Institute (CLSI) have to be done, also to establish especially normal ranges of the modified tests [49].

## Conclusions

In conclusion, an algorithm based on standard and modified thromboelastometric tests allows detection and differentiation of all DXaIs, DTI, VKA and DIL. A more complex algorithm, based on machine learning and decision-tree analysis, improves the accuracy of detection and differentiation to 98% compared to 94% with a simpler algorithm. Further validation of this algorithm in a prospective multicenter trial is needed.

## Supplementary Information

**Additional file 1: Supplemental Fig. 1.** Standard and new thromboelastometric tests are shown under control conditions, intake of anticoagulants and simulated dilutional coagulopathy. Depicted are median + IQR for coagulation time (CT; sec) or clot firmness amplitude 5 min after CT (A5; mm), respectively. A) CT_TFTEM_, B) A5_TFTEM,_ C) CT_ECATEM,_ D) CT-ratio TFTEM/ECATEM, E) CT_FIBTEM,_ and F) A5_FIBTEM_ are differentially altered following DTI (dabigatran), DXaIs, vitamin K antagonists or dilutional coagulopathy. *p < 0.05, **p < 0.01, ***p < 0.001, ****p < 0.0001; A5: clot firmness amplitude 5 min after CT; CT: coagulation time; DTI: direct thrombin inhibitor; DXaI: direct factor Xa inhibitor; IQR: interquartile range.

**Additional file 2: Table 1.** Patient’s characteristics. Data are presented as median (Q1/Q3) or proportion.

**Additional file 3: Supplemental Table 2.** thromboelastometric and standard laboratory variables of healthy volunteers and patients.

## Data Availability

The datasets used and/or analyzed during the current study are available from the corresponding author on reasonable request.
