# Supplementary figures and images for: Point-of-care detection and differentiation of anticoagulant therapy - development of thromboelastometry-guided decision-making support algorithms
Source: Thromb J. 2021 Sep 7;19:63. doi: 10.1186/s12959-021-00313-7 (PMC8425056; doi:10.1186/s12959-021-00313-7)

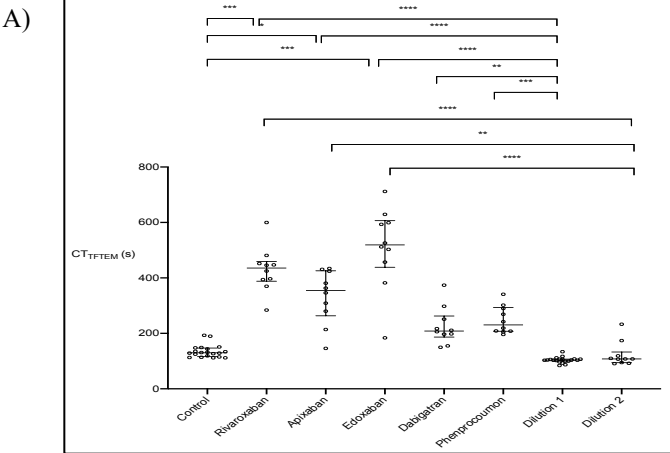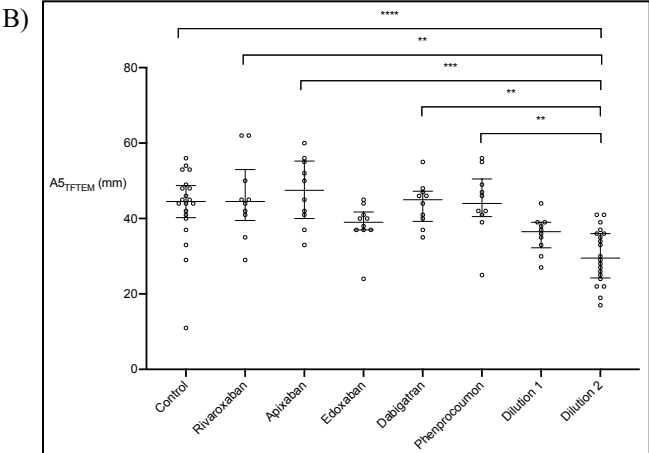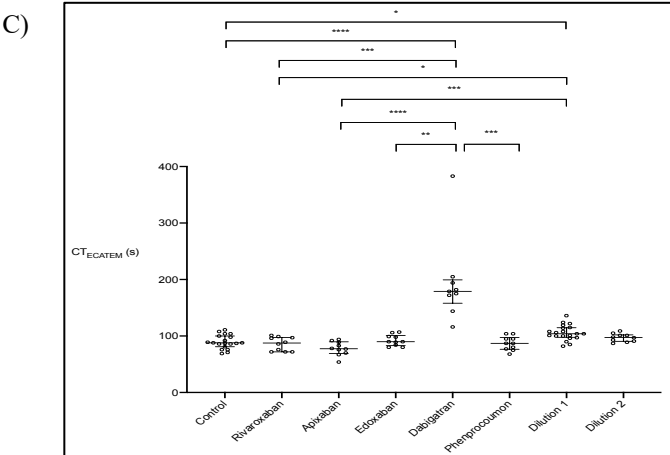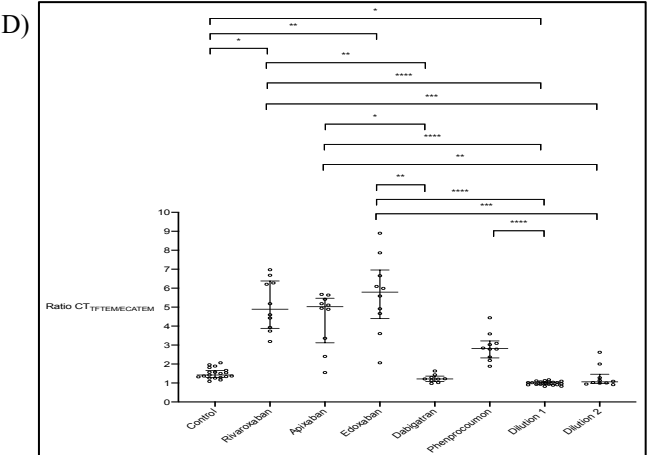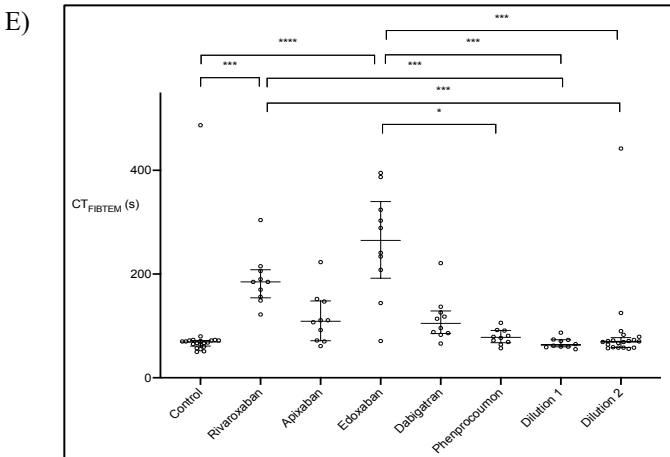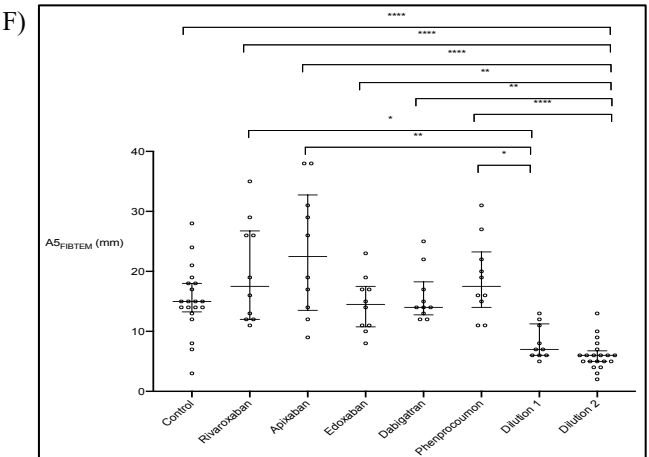

Supplement: Supplementary file 1 — Additional file 1: Supplemental Fig. 1. Standard and new thromboelastometric tests are shown under control conditions, intake of anticoagulants and simulated dilutional coagulopathy. Depicted are median + IQR for coagulation time (CT; sec) or clot firmness amplitude 5 min after CT (A5; mm), respectively. A) CTTFTEM, B) A5TFTEM, C) CTECATEM, D) CT-ratio TFTEM/ECATEM, E) CTFIBTEM, and F) A5FIBTEM are differentially altered following DTI (dabigatran), DXaIs, vitamin K antagonists or dilutional coagulopathy. *p < 0.05, **p < 0.01, ***p < 0.001, ****p < 0.0001; A5: clot firmness amplitude 5 min after CT; CT: coagulation time; DTI: direct thrombin inhibitor; DXaI: direct factor Xa inhibitor; IQR: interquartile range. [file 12959_2021_313_MOESM1_ESM.pdf]
