# Supplementary material for: Point-of-care detection and differentiation of anticoagulant therapy - development of thromboelastometry-guided decision-making support algorithms
Source: Thromb J. 2021 Sep 7;19:63. doi: 10.1186/s12959-021-00313-7 (PMC8425056; doi:10.1186/s12959-021-00313-7)
Supplement: Supplementary file 2 — Additional file 2: Table 1. Patient’s characteristics. Data are presented as median (Q1/Q3) or proportion. [file 12959_2021_313_MOESM2_ESM.docx]

Table 1: Patient’s characteristics. Data are presented as median (Q1/Q3) or proportion.

|  | Control  (n=20) | Dabigatran  (n=10) | Rivaroxaban  (n=10) | Apixaban  (n=10) | Edoxaban  (n=10) | Phenprocoumon  (n=10) |
| --- | --- | --- | --- | --- | --- | --- |
| **Gender** (female/male; n) | 13/7 | 7/3 | 6/4 | 4/6 | 5/5 | 4/6 |
| **Age** (years) | 62  (55/75) | 71  (65/76) | 63  (57/74) | 81  (76/83) | 80  (75/82) | 75  (57/79) |
| **Height** (cm) | 175  (166/180) | 178  (172/182) | 168  (164/171) | 171  (167/175) | 174  (162/177) | 174  (170/179) |
| **Body weight** (kg) | 78.5  (63.5/89.8) | 81.5  (70/102) | 72.5  (65.6/83.5) | 72.5  (69.5/83.2) | 86.5  (62/93) | 76.5  (61/85.5) |
| **Body mass index** (kg*m^2^) | 25.8  (22.4/27.8) | 24.9  (24.1/30.5) | 25.2  (22.6/27.2) | 25.3  (22.9/27.3) | 28.0  (23.9/30.4) | 25.4  (21.3/26.9) |
| **Creatinine serum concentration** (mg dl^-1^) | 1.0  (0.9/1.1) | 1.1  (1.1./1/3) | 1.2  (0.9/1.5) | 1.3  (1.1/1.4) | 1.1  (1.0/1.3) | 1.2  (1.1/1.7) |
| **Albumin serum concentration** (g dl^-1^) | 4.3  (4.1/4.5) | 4.3  (3.5/4.5) | 3.8  (3.5/4.0) | 3.5  (3.2/3.8) | 3.8  (3.6/4.0) | 3.9  (3.6/4.2) |
| **GFR (**ml min^-1^) | 92  (79/97) | 85  (61/101) | 72  (55/98) | 57  (53/83) | 74  (52/83) | 55  (40/87) |
| **Hemoglobin** (g dl^-1^) | 14.2  (12.8/15.1)  Dilution 1  6.1  (4.8/6.9)  Dilution 2  9.9  (9.1/10.3) | 14.0  (13.6/15.3) | 11.7  (10.2/13.1) | 12.2  (9.5/13.7) | 12.3  (11.3/13.6) | 12.2  (10.6/12/6) |
| **Red blood cells** (10^12^ L^-1^) | 4.6  (4.4/5.1) | 4.5  (3.8/5.0) | 4.0  (3.8/4.4) | 3.9  (3.5/4.6) | 4.1  (3.8/5.0) | 4.2  (3.8/4.6) |
| **Hematocrit** (%) | 41  (37/44) | 41  (40/44) | 35  (32/40) | 36  (29/40) | 37  (33/41) | 36  (33/38) |
| **Platelets** (10^9^ L^-1^) | 227  (14/297) | 214  (179/265) | 252  (158/346) | 257  (194/376) | 176  (136/250) | 217  (130/252) |

GFR: Glomerular filtration rate
