# Supplementary material for: Point-of-care detection and differentiation of anticoagulant therapy - development of thromboelastometry-guided decision-making support algorithms
Source: Thromb J. 2021 Sep 7;19:63. doi: 10.1186/s12959-021-00313-7 (PMC8425056; doi:10.1186/s12959-021-00313-7)
Supplement: Supplementary file 3 — Additional file 3: Supplemental Table 2. thromboelastometric and standard laboratory variables of healthy volunteers and patients. [file 12959_2021_313_MOESM3_ESM.docx]

|  | Controls | Dabigatran | Rivaroxaban | Apixaban | Edoxaban | Phenprocoumon | Dilution 1 | Dilution 2 |
| --- | --- | --- | --- | --- | --- | --- | --- | --- |
| CT_NATEM_  (s) | 666  (574/723) | 966  (828/1217) | 739  (610/955) | 487  (458/626) | 861  (673/1206) | 671  (570/849) | 717  (676/859) | 649  (600/700) |
| CT_EXTEM_  (s) | 65  (61/72) | 118  (92/141) | 189  (160/218) | 109  (72/133) | 269  (198/295) | 87  (75/105) | 61  (56/75) | 77  (66/84) |
| CT_FIBTEM_  (s) | 69  (61/72) | 105  (85/129) | 185  (154/208) | 109  (72/148) | 265  (192/340) | 78  (68/91) | 64  (60/73) | 70  (58/78) |
| CT_INTEM_  (s) | 197  (177/204) | 302  (211/375) | 280  (206/306) | 196  (174/224) | 293  (207/317) | 198  (178/238) | 207  (188/216) | 214  (198/238) |
| CT_HEPTEM_  (s) | 191  (157/206) | 314  (228/389) | 219  (197/248) | 206  (182/262) | 302  (221/341) | 201  (183/225) | 204  (185/219) | 205  (183/229) |
| CT_TFTEM_  (s) | 130  (116/147) | 209  (187/263) | 436  (388/459) | 355  (264/426) | 519  (438/607) | 231  (208/293) | 108  (94/133) | 104  (100/107) |
| CT_ECATEM_  (s) | 88  (81/100) | 179  (158/200) | 88  (72/98) | 78  (69/90) | 90  (83/101) | 87  (77/97) | 98  (91/102) | 104  (98/115) |
| A5_NATEM_ (mm) | 28  (21/34) | 19  (16/24) | 30  (27/47) | 34  (21/42) | 24  (18/34) | 33  (26/39) | 23  (20/27) | 22  (19/28) |
| A5_EXTEM_ (mm) | 46  (42/54) | 49  (45/53) | 53  (46/62) | 53  (46/64) | 45  (41/50) | 49  (43/61) | 33  (29/37) | 29  (24/34) |
| A5_FIBTEM_ (mm) | 15  (13/18) | 14  (13/18) | 18  (12/27) | 23  (14/33) | 15  (11/18) | 18  (14/23) | 7  (6/11) | 6  (5/7) |
| A5_INTEM_ (mm) | 46  (41/52) | 46  (39/51) | 50  (45/55) | 49  (42/61) | 45  (41/51) | 47  (41/56) | 34  (31/37) | 30  (26/36) |
| A5_HEPTEM_ (mm) | 47  (42/51) | 46  (38/47) | 52  (44/60) | 45  (39/58) | 44  (40/47) | 46  (40/58) | 35  (33/38) | 30  (25/38) |
| A5_TFTEM_ (mm) | 45  (40/49) | 45  (39/47) | 45  (40/53) | 48  (40/55) | 39  (37/42) | 44  (41/51) | 37  (32/39) | 30  (24/36) |
| A5_ECATEM_ (mm) | 49  (46/53) | 46  (43/51) | 56  (48/62) | 54  (45/62) | 46  (42/53) | 49  (42/58) | 39  (33/40) | 34  (29/38) |
| INR | 1.0  (1.0/1.0) | 1.2  (1.1/1.3) | 1.3  (1.2/1.4) | 1.2  (0.98/1.3) | 1.5  (1.3/1.6) | 1.9  (1.8/2.6) | 1.5  (1.5/1.7) | 1.5  (1.4/1.7) |
| aPTT (s) | 26  (24/28) | 38  (36/47) | 32  (30/34) | 27  (25/31) | 31  (27/39) | 35  (30/39) | 43  (40/51) | 44  (37/52) |
| TT (s) | 18  (17/19) | 150  (136/150) | 17  (16/18) | 17  (17/19) | 19  (17/19) | 18  (18/19) | 22  (20/24) | 21  (20/23) |
| DOAC plasma conentration ng/ml | / | 80  (78/98) | 178  (143/282) | 133  (89/176) | 257  (139/377) | / | / | / |

Supplemental table 2: thromboelastometric and standard laboratory variables of healthy volunteers and patients.

CT: clotting time; TT: thrombin time, aPTT: partial thromboplastin time; DOAC: direct oral anticoagulant
